# Small nucleolar RNA U91 is a new internal control for accurate microRNAs quantification in pancreatic cancer

**DOI:** 10.1186/s12885-015-1785-9

**Published:** 2015-10-24

**Authors:** Alexey Popov, Arpad Szabo, Václav Mandys

**Affiliations:** Department of Pathology, Third Faculty of Medicine, Charles University in Prague and University Hospital Kralovske Vinohrady, Srobarova 50, 100 00, Prague 10, Czech Republic

**Keywords:** miRNA, Pancreatic cancer, Internal control, RT-qPCR (reverse transcription quantitative PCR), Pancreatic ductal adenocarcinoma

## Abstract

**Background:**

RT-qPCR quantification of miRNAs expression may play an essential role in pancreatic ductal adenocarcinoma (PDAC) diagnostics. RT-qPCR-based experiments require endogenous controls for the result normalization and reliability. However, expression instability of reference genes in tumors may introduce bias when determining miRNA levels.

**Methods:**

We investigated expression of 6 miRNAs, isolated from FFPE samples of pancreatic adenocarcinomas. Four internal controls were utilized for RT-qPCR result normalization: artificial miR-39 from C. elegans, U6 snRNA, miR-16 and snoRNA U91.

**Results:**

We found miR-21, miR-155 or miR-217 expression values in tumors may differ up to several times, depending on selected internal controls. Moreover, different internal controls can produce controversial results for miR-96, miR-148a or miR-196a quantification. Also, expression of our endogenous controls varied significantly in tumors. U6 demonstrated variation from −1.03 to 8.12-fold, miR-16 from −2.94 up to 7.38-fold and the U91 from −3.05 to 4.36-fold respectively. On the other hand, the most stable gene, determined by NormFinder algorithm, was U91. Each miRNA normalized relatively to the spike or U91, demonstrated similar expression values. Thus, statistically significant and insignificant differences between tumors and normal tissues for miRNAs were equal for the spike and the U91. Also, the differences between the spike and U91 were statistically insignificant for all of miRs except miR-217. Among three endogenous controls, U91 had the lowest average expression values and standard deviation in cancer tissues.

**Conclusions:**

We recommend U91 as a new normalizer for miRNA quantification in PDACs.

## Background

Pancreatic ductal adenocarcinoma (PDAC) is the most common and the most aggressive primary pancreatic neoplasm. The majority of patients are diagnosed by the time the tumor had already invaded peripancreatic structures or has metastasized [1]. Therefore, there is a need for biomarkers enabling early detection of asymptomatic PDACs. miRNAs are stable in tissues and blood plasma [2]; consequently they are ideal molecules to be utilized as biomarkers. miRNAs are involved in oncogenesis, apoptosis and cell growth; thereby functioning as tumor suppressors or oncogenes [3–6]. A large number of miRNAs are proven to be overexpressed in pancreatic cancer [7–11]. On the other hand, the expression of miRNA-coding genes, which act as tumor suppressors, could be inhibited in cancer cells [12–16]. Alterations in the miRNAs expression profile of cancer in comparison with normal tissues could be used in pancreatic cancer diagnostics. The high sensitivity of reverse transcription quantitative PCR (RT-qPCR) has made it a popular method in the measurement of tumor miRNA expression. RT-qPCR-based experiments require endogenous controls for result normalization, reliability and reproducibility. The endogenous control helps to correct differences between sample quality and variations during RNA extraction or reverse transcription procedures. Housekeeping genes, ribosomal, small nuclear or nucleolar RNAs can play the role of such internal controls. However, according to experimental data, expression levels of these genes may differ in neoplastic and normal tissues [17–19]. These variations may introduce bias to experiment results.

In this study we compared the expression of selected miRNAs in samples from pancreatic cancer and normal pancreatic parenchyma and evaluated the influence of different internal controls on the expression data alterations.

## Methods

### Patients and tissue specimens

FFPE blocks of pancreatic ductal adenocarcinomas were retrieved from the archive of the Department of Pathology of the 3^rd^ Faculty of Medicine of the Charles University and University Hospital Kralovske Vinohrady in Prague. The samples were collected from 24 patients, who had undergone pancreatoduodenectomy, distal pancreatectomy or total pancreatectomy between 2007 and 2012. Participants signed a written informed consent before the study. The study was performed according to the Declaration of Helsinki and approved by the Ethics Committee of the Third Faculty of Medicine (Charles University in Prague, Czech Republic). The resolution 1006/2012 was signed by Dr. Marek Vacha, Ph.D, Head of the Ethics Committee.

In the selected FFPE blocks the tumor occupied the majority of the slide. As negative control, FFPE blocks containing normal pancreatic tissue of the respective patients were selected.

### Clinicopathological features

The age of patients with resected pancreatic adenocarcinoma ranged from 36 to 83 years, with a median of 65.5 years. In total, 11 patients were women and 13 patients were men. Genetic syndromes were described in none of the patients. Grossly, 18 tumors were located in the head of the pancreas, 1 in the body of the pancreas and 5 in the tail of the pancreas.

The tumors showed in all of the selected cases the features of conventional ductal pancreatic adenocarcinoma. According to the guidelines of the WHO Classification of Tumors of the Gastrointestinal Tract, 3^rd^ and 4^th^ edition, 1 tumor was well differentiated, 14 tumors were moderately differentiated and 9 tumors were poorly differentiated. In one patient, a synchronous mucinous cystic neoplasm (MCN) was identified in the cauda of the pancreas. In another patient the tumor originated from an MCN. In 3 patients the resected tumor was described as pT1, in 5 patients pT2, in 15 patients pT3 and in one patient pT4. Additionally, lymph node metastases were confirmed in the resected specimens of 18 patients.

### RNA isolation and reverse transcription

One to three 6 μm thick unstained paraffin embedded tissue sections were procured for RNA extraction, using the miRNeasy FFPE kit (Qiagen), following the manufacturer’s instructions. Two microliters of isolated RNA were used for RNA quantity and purity analysis. Optical density at 260 and 280 nm was measured with a multi-detection microplate reader Synergy HT (BioTek), including Take3 micro-volume plate. RNA integrity was assessed with denaturing agarose gel electrophoresis and GeneTools 3.08 software (SynGene).

A mix of 10 stem-loop primers was used for miRNA reverse transcription. Stem-loop primers were selected for the analysis, because their structure reduces annealing of the primer to pre- and pri-miRNAs, therefore increasing the specificity of the assay. Primers were designed with miRNA primer designer software, kindly provided by Dr. Fuliang Xie, East Carolina University. The stem-loop primer sequences for the internal controls, including the alien spike (miR-39 from C. elegans), and the examined pancreatic miRNAs are listed in Tables 1 and 2. The spike RNA was added to the reaction mix directly before the reverse transcription. Alien spike can’t be used as a normalizer for differences between samples during the RNA isolation, because tissue sections may contain different amounts of tissue. Therefore, the addition of spike before RNA isolation may introduce bias, because a ratio between amount of the RNA and the alien spike concentration may vary from sample to sample.

**Table 1:** Stem-loop primers for the internal controls

Control name	Stem-loop primer sequence
mir-39 C. elegance	GTCGTATCCAGTGCAGGGTCCGAGGTATTCGCACTGGATACGACTATTAC
U6	GTCGTATCCAGTGCAGGGTCCGAGGTATTCGCACTGGATACGACAAAAATATGG
U91 snoRNA	GTCGTATCCAGTGCAGGGTCCGAGGTATTCGCACTGGATACGACCGGCCT
miR-16	GTCGTATCCAGTGCAGGGTCCGAGGTATTCGCACTGGATACGACCGCCAA

**Table 2:** Stem-loop primers for the miRNAs

miRNA name	Stem-loop primer sequence
mir-21	GTCGTATCCAGTGCAGGGTCCGAGGTATTCGCACTGGATACGACTCAACA
miR-96	GTCGTATCCAGTGCAGGGTCCGAGGTATTCGCACTGGATACGACAGCAAAAATGTG
miR-148a	GTCGTATCCAGTGCAGGGTCCGAGGTATTCGCACTGGATACGACAGTCGGAG
miR-155	GTCGTATCCAGTGCAGGGTCCGAGGTATTCGCACTGGATACGACACCCCTATCACG
miR-196a	GTCGTATCCAGTGCAGGGTCCGAGGTATTCGCACTGGATACGACCCCAACAACATG
miR-217	GTCGTATCCAGTGCAGGGTCCGAGGTATTCGCACTGGATACGACTCCAATCAGTTC

Reverse transcription was carried out, using RevertAid Reverse Transcriptase (Thermo Scientific), in a 50 μl reaction mixture, containing the following reagents: 1 μg of DNA-free RNA, reaction buffer [50 mM Tris–HCl (pH 8.3 at 25 °C), 50 mM KCl, 4 mM MgCl_2_ and 50 mM DTT]; 1 mM of dATP, dTTP, dCTP, dGTP; 20 IU rRNasin ribonuclease inhibitor; 100 IU of moloney murine leukemia virus reverse transcriptase (M-MuLV RT) and the primer mix, including 20 pmol of each stem-loop primer. Artificial spike RNA (miR-39 from C. elegans, 5 × 10^8^ copies) was also added to the reaction as internal control. After initial denaturation (5 min at 70 °C, then cooling samples on ice), the reactions were incubated at 25 °C (10 min), and then at 42 °C for 1 h. To stop the reaction, the mixture was heated at 70 °C for 10 min.

### Real-time qPCR

cDNA samples were amplified in duplicates, using the Applied Biosystems 7500 Fast real-time PCR system and Hot FirePol EvaGreen qPCR Mix Plus (Solis BioDyne). The reaction mix included 10 pmol of each primer (miRNA specific and the universal (Table 3)) and 2 μl of cDNA.

**Table 3:** Real-time qPCR primers

Primer name	Primer sequence
Universal primer	ATCCAGTGCAGGGTCCGAGG
mir-39 C. elegance	GCGGCGGAGCTGATTTCGTCTTG
U6	GCGGCGGCGCAAGGATGACACG
U91 snoRNA	GCGGCGGTGGCCGATGATGACG
miR-16	GCGGCGGTAGCAGCACGTAAAT
mir-21	GCGGCGGTAGCTTATCAGACTG
miR-96	GCGGCGGTTTGGCACTAGCAC
miR-148a	GCGGCGGAAAGTTCTGAGACACTCC
miR-155	GCGGCGGTTAATGCTAATCGTG
miR-196a	GCGGCGGTAGGTAGTTTCATGTTG
miR-217	GCGGCGGTACTGCATCAGGAAC

Amplification of the cDNAs was performed at the following thermal conditions: denaturation at 94 °C for 15 min, followed by 40 cycles consisting of denaturation at 94 °C for 15 s, annealing at 48 °C for 60 s and DNA synthesis at 72 °C for 40 s. Reaction product specificity was controlled with their respective melting curves.

### Statistical analysis

The expression of miRNAs in neoplastic and normal tissues was compared utilizing a paired two-tailed Student’s *t* test as well as a one-way ANOVA analysis. *P*-values below 0.05 were regarded as statistically significant. RT-qPCR data (threshold cycles) were linearized, and the NormFinder algorithm was used to calculate the most stable gene among the internal controls.

## Results

### Evaluation of miRNA expression levels in PDAC samples

We investigated the expression of 6 miRNAs isolated from FFPE samples of pancreatic adenocarcinomas from 24 patients. The following microRNAs were selected: miR-21, which promotes cell proliferation and may accelerate tumorigenesis [8, 9, 20]; miR-155, which interacts with TP53 INP1 and transforming growth factor β (TGF-β) [11, 21, 22]; miR-96 and miR-217, which may act as a tumor suppressors, inhibiting the KRAS-signaling pathway [13, 14] also miR-148a and miR-196a, which are frequently included in experimental panels for pancreatic carcinoma diagnosis [23–29].

**Fig. 1:**
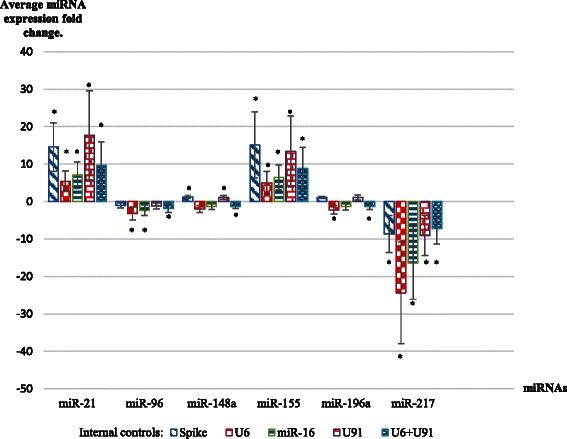
Average expression of six miRNAs in pancreatic cancers. Four different internal controls and one combination of two of them (U6 + U91) were used for the results normalization. Data are presented as mean ± standard deviation (SD). Statistically significant differences (Student’s *t*-test, *p* < 0.05) between tumors and normal tissues are labeled with asterisk. MicroRNA expression values depend on the selected internal control and may vary up to several times

Four internal controls were utilized for qRT-PCR result normalization: an alien spike (artificial miR-39 from C. elegans) and three endogenous controls – U6 snRNA, miR-16 and snoRNA U91. miRNA expression values were normalized relative to each of these controls, and significant variations for the same miRNAs were found (Fig. 1, Table 4). In comparison with normal pancreatic tissue, miR-21 was significantly overexpressed, up to 14.56-fold (*p* < 0.01) in the case of the alien spike. However, for other internal controls, fold change values were shifted to 5.44 for U6 (*p* < 0.01), 7.03 for miR-16 (*p* < 0.01) and 17.71 for U91 (*p* < 0.01), respectively (Table 4, Fig. 1). The miR-155 also demonstrated increased expression levels with great variations between internal controls: 15.1-fold for the spike (*p* < 0.01); 5.05-fold for U6 (*p* < 0.01); 6.39-fold for miR-16 (*p* < 0.01) and 13.36-fold for U91 (*p* < 0.01). miRNA miR-96 in pancreatic carcinoma did not show significant differences in comparison with normal tissues, when normalizing to the alien spike (−1.04-fold, *p* > 0.05), as well as to U91 (−1.17-fold, *p* > 0.05). But, this miRNA was significantly down-regulated, when the expression was measured relative to U6 (−3.22-fold, *p* < 0.01) or miR-16 (−2.32-fold, *p* < 0.01). Also, no significant differences were found for miR-148a, normalized to spike (1.25 fold, *p* > 0.05) and U91 (1.06, *p* > 0.05). But, this miRNA was significantly inhibited for U6 (−1.33 fold, *p* < 0.01) and miR-16 (−2.04 fold, *p* < 0.01). Expression of miR-196a was slightly up-regulated relatively the alien spike (1.09-fold) and U91 (1.13-fold), without the results being statistically significant (*p* > 0.05). On the other hand, miR-196a was significantly down-regulated for U6 (−2.22-fold, *p* < 0.01), as well as not statistically significant for the miR-16 (−1.35, *p* > 0.05). The expression of miR-217 was significantly lower in all PDACs than in normal pancreatic tissues for all the examined internal controls (*p* < 0.01) (Table 4, Fig. 1).

**Table 4:** Average miRNAs fold change values in pancreatic cancers in comparison with normal tissues

miRNAs	Internal controls				
	Spike	U6	miR-16	U91	U6 + U91
miR-21	14.56 ± 6.468; ***p*** **< 0.01**	5.44 ± 2.73; ***p*** **< 0.01**	7,03 ± 3,614; ***p*** **< 0.01**	17.71 ± 11.922; ***p*** **< 0.01**	9.67 ± 6.287; ***p*** **< 0.01**
miR-96	−1.04 ± 0.668; *p* > 0.05	−3.22 ± 1.766; ***p*** **< 0.01**	−2,32 ± 1.376; ***p*** **< 0.01**	−1.17 ± 0.831; *p* > 0.05	−1.85 ± 1.134; ***p*** **< 0.01**
miR-148a	1.25 ± 0.429; *p* > 0.05	−2.04 ± 0.92; ***p*** **< 0.01**	−1.33 ± 0.782; ***p*** **< 0.05**	1.06 ± 0.549; *p* > 0.05	−1.27 ± 0.594; *p* > 0.05
miR-155	15.1 ± 8.786; ***p*** **< 0.01**	5.05 ± 2.992; ***p*** **< 0.01**	6.39 ± 3.312; ***p*** **< 0.01**	13.36 ± 9.477; ***p*** **< 0.01**	8.79 ± 5.675; ***p*** **< 0.01**
miR-196a	1.09 ± 0.306; *p* > 0.05	−2.22 ± 1.09; ***p*** **< 0.01**	−1.35 ± 0.905; *p* > 0.05	1.13 ± 0.676; *p* > 0.05	−1.34 ± 0.726; ***p*** **< 0.05**
miR-217	−8.69 ± 4.99; ***p*** **< 0.01**	−24,39 ± 13.616; ***p*** **< 0.01**	−16.39 ± 9.71; ***p*** **< 0.01**	−9.09 ± 5.323; ***p*** **< 0.01**	−7.19 ± 4.161; ***p*** **< 0.01**

MicroRNAs expression was measured relative to four different internal controls and combination of two of them (U6 + U91). Negative fold change values indicate downregulation of the miRNAs in cancer samples. Data are presented as means ± standard deviation (SD). *p* < 0.05 is considered as statistically significant. *P* values of the Student’s *t*-test for the significant differences are shown in bold

NormFinder algorithm was used to calculate the most stable pairing of internal controls. According to our results, the best combination was U6 and U91 (stability value = 0036; Table 5) for the miRNAs expression evaluation. Normalized to this most stable pair, miRNAs miR-21 and miR-155 demonstrated significant upregulation (9.67-fold and 8.79-fold, *p* < 0.01). Activity of miR-96, miR-196a and miR-217 was significantly inhibited (−1.85-fold, −1.34-fold and −7.19-fold, *p* < 0.01). The miR-148a expression also was down-regulated, but the decrease was not statistically significant (−1.27-fold, *p* > 0.05).

**Table 5:** Stability evaluation of all internal controls using NormFinder algorithm

Gene name	Stability value		Best gene: U91
Spike	0.085		Stability value: 0.056
U6	0.056		
miR-16	0.078		Best combination of two genes: U6 and U91
U91	0.056		Stability value for best combination of two genes: 0.036
Intragroup variation			
Group identifier	Normal	PDACs	
Spike	0.061	0.065	
U6	0.013	0.026	
miR-16	0.023	0.086	
U91	0.017	0.053	
Intergroup variation			
Group identifier	Normal	PDACs	
Spike	0.054	−0.054	
U6	−0.026	0.026	
miR-16	−0.043	0.043	
U91	0.016	−0.016	

### Determination of the best normalizers for the miRNAs expression measuring

To find the best normalizer, we compared miRNA expression levels, normalized relatively the artificial spike and other endogenous controls, including the combination of U6 + U91, with one-way ANOVA analysis for each individual miRNA. The null hypothesis (H_o_) was, that average fold change values, calculated for the each individual miRNA, are the same for all the internal controls. However, the differences were significant in the case of miR-21, miR-96, miR-148a, miR-155 and miR-196a (*p* < 0.01). For miR-217, the difference was not statistically significant (*p* > 0.05). Consequently, we compared miRNA expression normalized to the spike, with their normalization to the other individual examined endogenous controls, using a paired two-tailed Student’s *t*-test. Differences of miRNA expression, normalized to U6 or the combination of U6 and U91, in comparison with the alien spike, were statistically significant for all miRNAs (*p* < 0.01; Table 6). In the case of miR-16, the difference was not significant for miR-217 only (*p* > 0.05; Table 6). On the other hand, the difference between spike and the U91 was statistically insignificant for all miRNAs (*p* > 0.05; Table 6), except for miR-217 (*p* < 0.05; Table 6). Thus, one endogenous control was found which demonstrated a behavior very similar to the alien spike.

**Table 6:** The difference between normalizers

miRNAs	Internal controls			
	U6	miR-16	U91	U6 + U91
miR-21	2.97E-06; ***p*** **< 0.001**	0.003404; ***p*** **< 0.01**	0.7979; *p* > 0.05	0.000789; ***p*** **< 0.01**
miR-96	0.003078; ***p*** **< 0.01**	0.02723; ***p*** **< 0.05**	0.639203; *p* > 0.05	0.038631; ***p*** **< 0.05**
miR-148a	4.66E-06; ***p*** **< 0.001**	0.007849; ***p*** **< 0.01**	0.3078; *p* > 0.05	0.000586; ***p*** **< 0.01**
miR-155	6.13E-05; ***p*** **< 0.01**	0.000873; ***p*** **< 0.01**	0.534202; *p* > 0.05	0.001831; ***p*** **< 0.01**
miR-196a	7.93E-07; ***p*** **< 0.001**	0.042034; ***p*** **< 0.05**	0.862491; *p* > 0.05	0.000117; ***p*** **< 0.01**
miR-217	0.003951; ***p*** **< 0.01**	0.065696; *p* > 0.05	0.024668; ***p*** **< 0.05**	0.008373; ***p*** **< 0.05**

MicroRNA expression levels were normalized relative to the alien spike, in comparison with the normalizations relative to other internal controls. Data are presented as *P*-values of the paired Student’s *t*-test. *p* < 0.05 was considered statistically significant. *P*-values for the statistically significant differences are shown in bold

We investigated the causes of miRNA expression variations and their dependence on certain normalizers, and thus attempted to find the most suitable normalizer. The 2^-ΔΔCT^ method was used for the miRNAs expression quantification, where CT is cycle threshold and ΔΔCT = ((CT_miRNA_)_tumor_-(CT_control_)_tumor_)-((CT_miRNA_)_normal_-(CT_control_)_normal_) For accurate miRNA quantification, in theory, CT values for the internal control gene should be very close for tumors and normal tissues, ideally (CT_control_)_tumor_ = (CT_control_)_normal._ However, this CT_control_ value may be shifted up to several cycles in tumors (for example, up to ± n cycles), if the expression of endogenous control differs in tumor and normal tissue. This difference may introduce a bias to the miRNA fold change calculations:

$$ \Delta \Delta \mathrm{CT} = \left({\left(\mathrm{C}{\mathrm{T}}_{\mathrm{miRNA}}\right)}_{\mathrm{tumor}}\hbox{--} \left({\left(\mathrm{C}{\mathrm{T}}_{\mathrm{control}}\right)}_{\mathrm{normal}} \pm \mathbf{n}\right)\right)-\left({\left(\mathrm{C}{\mathrm{T}}_{\mathrm{miRNA}}\right)}_{\mathrm{normal}}\hbox{--} {\left(C{\mathrm{T}}_{\mathrm{control}}\right)}_{\mathrm{normal}}\right). $$

While analyzing the amplification curves of the different internal controls, almost in all tumor samples a cycle threshold (CT) shift of *n* = 5 or even 6 cycles upwards in comparison with the normal tissue was apparent (Table 7). For example, CT values of the spike were very similar for PDAC and the normal tissues, they differed less than *n* = 0.8 cycle (Table 7). Nevertheless, for other normalizers these values varied from *n* = −6.20 up to *n* = 5.8 cycles (Table 7). We measured expression levels of our endogenous controls, using the alien spike for normalization. As expected, U6 expression in tumors varied from −1.03 to 8.12-fold, miR-16 showed variations from −2.94 up to 7.38-fold in different tumors, and the U91 from −3.05 to 4.36-fold respectively. The difference in expression was statistically significant for all endogenous controls (*p* < 0.01; Table 8). Also, U6 was overexpressed in 22 tumors from 24, miR-16 in 18 tumors and U91 in 14 correspondently. miR-16 was downregulated in 6 tumors and U91 in 5 tumor samples (Table 9). Thus, all selected endogenous controls demonstrated expression instability in tumor samples.

**Table 7:** Cycle threshold values (CT) for endogenous controls are different in tumors and normal tissues

CT values shift (*n*) between tumors and normal tissues	Internal controls			
	Spike	U6	miR-16	U91
Min	0,61	5,02	5,80	1,90
Max	−0,70	−5,79	−6,20	−2,06
Average ± SD	−0,13 ± 0,251	−1,23 ± 1,127	−0,78 ± 1,315	−0,36 ± 0,833

These values are often shifted ± *n* cycles in tumors. It means that expression of endogenous control genes can vary in tumors

**Table 8:** Expression values of candidate endogenous control genes are highly variable in PDACs in comparison with normal tissues

Fold change values in PDACs, measured relative to the spike	Genes		
	U6	miR-16	U91
Min	−1.03	−2.94	−3.05
Max	8.12	7.38	4.36
Average ± SD	3.13 ± 1.331; *p* < 0.01	2.69 ± 1.540; *p* < 0.01	1.61 ± 0.730; *p* < 0.01

*P* values of the Student’s *t*-test, when *p* < 0.05, were considered statistically significant

**Table 9:** Expression of the endogenous controls is unstable in all tumor samples

Expression of the endogenous controls in tumors	Endogenous controls		
	U6	miR-16	U91
Upregulated	22	18	14
Downregulated	0	6	5
Close to normal tissues	2	0	6
Total number of patients	24		

To identify the most stable internal control, the NormFinder algorithm was used. RT-qPCR expression data for all internal controls were linearized and compared in two groups: tumors and normal pancreatic tissues (Table 5). The most stable gene was U91 (stability value = 0.056), but stability values for all internal controls were close (0.085; 0.056 and 0.078 for the spike, U6 and miR-16, respectively; Table 5). U6 had the same stability value as U91 (0.056), but it demonstrated higher levels of intergroup variation (Table 5). Surprisingly, the most unstable control was the artificial spike (0.085; Table 5). The NormFinder can calculate variations between two groups, including all normal or cancer samples, but it is unable to evaluate the differences between normal and cancer tissues among individual patients. This may be the reason for the “instability” of the alien spike. The most stable pair of internal controls was a combinations of two genes, U6 and U91 (stability value = 0.036; Table 5).

## Discussion

### MicroRNA expression values depend on a selected internal control

Pancreatic ductal adenocarcinoma is one of the most frequently occurring solid cancers and it carries an extremely poor prognosis. As such, an extensive search for biomarkers of early disease is undergoing, miRNAs may have the ideal characteristics to fulfil this role. Due to their stability and resistance against RNase degradation, they are viable in a wide range of samples. Viable miRNAs for PDAC diagnosis may be isolated from frozen and paraffin embedded tissue samples, stool, blood plasma, or pancreatic juice, [24, 28, 30, 31].

For our analysis we have selected miRNAs, frequently described to be dysregulated in various types of PDACs samples. Studies mapping microRNA expression using microarrays have proven considerable heterogeneity in pancreatic carcinomas. Zhang et al. have demonstrated relative expression values miRNAs spanning 6-logs (from 0.01–10,000) among individual cases [27]. Among tumor samples we determined up to 45-fold variability in both miR-21 and miR-155 levels. During our brief review of literature we have noticed that the mean values of miRNA-levels in tumors varied among studies. There are many factors including differences in reagents/materials for miRNAs quantification protocols and data-processing algorithms, which can contribute to the variation. One of these factors is a variety of controls, which were used for normalization. Thus, the differences in the mean expression of miRNAs may be at least partially explained by the choice of controls for normalization.

For example, when normalizing with snoRNA U6, Bloomston et al. measured a median 2.2-fold increase in tumor miR-21 levels [24]. Zhang et al., using the same internal control, found that expression of miR-21 was up-regulated up to 6888-fold in several tumors [27]. Hong et al. reported about up to 550-fold increase in PDACs, normalizing relative to U6 [31]. When using both U6 and 5S as endogenous controls, du Rieu et al. detected a 20.1-fold tumor miR-21 up-regulation [8]. In our study, when normalizing with U6, a mean 5.5-fold increase in miR-21 in tumors was present. However, when normalizing to miR-16 a 7.03-fold increase was present (*p* < 0.01, Table 4). Wang et al. detected in plasma samples with miR-16 only a mean 2.42-fold up-regulation [30]. On the other hand, when normalizing to the artificial spike or with U91 we detected a mean 14.56-fold and 17.71-fold increase (*p* < 0.01, Table 4).

The data about miR-96 expression in PDAC are controversial. Several groups of authors reported about miR-96 expression fold increase in experiments with microarray [24, 32]. For example, Bloomston et al. measured an average 1.77-fold increase, when determining miR-96 levels in PDACs [24]. Kent et al. also demonstrated 2.7-fold upregulation of miR-96 in pancreatic cancer cell lines [32]. On the other hand, miR-96 has been shown to be frequently down-regulated in experiments, utilizing Northern blot or RT-qPCR [13, 15, 25, 31, 33]. Szafranszka et al. determined in their study a −4.35-fold decrease in tumor miR-96 expression, when normalizing to miR-24 [25]. Hong et al. as well as Feng et al. showed that miR-96, normalized to U6, was downregulated in PDAC samples up to −8-fold [31, 33]. With U6, miR-16 and combination of U6 + U91 respectively, we demonstrated a statistically significant mean −3.22-fold, −2.32-fold and −1.85-fold decrease in tumor tissue (*p* < 0.01, Table 4). However, expression analysis with the artificial spike and U91 alone yielded a statistically insignificant alteration in miR-96 expression in tumors in comparison with normal tissues (*p* > 0.05, Table 4).

miRNA miR-148a expression is described to be down-regulated in PDAC due to promoter hypermethylation, which represents an early event in pancreatic carcinogenesis [15]. Bloomston et al. as well as Jamieson et al. measured an average −5.5-fold and −7.14-fold decrease respectively, when determining miR-148 expression with a microarray [24, 29]. In experiments, utilizing RT-qPCR, Szafranszka et al. demonstrated −6.15-fold decrease of miR-148a levels, with miR-24 as normalizer [25]. However, Ma et al. and Zhang et al., normalizing to U6, determined a respective −2.86-fold and −2.5-fold downregulation in PDAC samples [34, 35]. Hanoun et al. also reported about miR-148a down-regulation, using U6 like the endogenous control [15]. In our study, tumor miR-148a levels were −2.04-fold and −1.33-fold decreased with U6 and miR-16 as a normalizers, respectively (*p* < 0.01 and *p* < 0.05, Table 4). On the other hand, analysis of miR-148a expression, normalized to the alien spike, U91 and a combination of U6 and U91 did not determine statically significant differences in expression between cancerous and non-cancerous tissues (*p* > 0.05, Table 4).

The miR-155 is an onco-miR, overexpressed in early pancreatic adenocarcinoma precursors and invasive PDAC [11]. The miR-155 expression in PDACs and pancreatic cancer cell lines, measured by microarray, ranged from 1.8 to 2.9-fold in different studies [24, 28, 29, 36, 37]. On the other hand, Habbe et al. found a mean 11.6-fold increase in intraductal papillary mucinous neoplasms, which was measured by RT-qPCR relative to U6 [11]. Zhang et al. also used U6 like a normalizer in their study. They reported about up to 52-fold increase in individual cases [27]. In our pancreatic carcinomas a mean 5.05-fold increase was present, when normalizing to U6 (*p* < 0.01, Table 4). However, Ma et al. measured only a 2.11-fold increase with the same endogenous control [34]. Wang et al. determined a 3.74-fold increase in serum miR-155 levels in cancer, when normalizing with miR-16 [30]. Our PDAC samples showed, on the other hand, a mean 6.39-fold increase with miR-16 as internal control (*p* < 0.01, Table 4). However, the expression levels were several times higher, measured relative to the alien spike or U91 - 15.1 and 13.36-fold respectively (*p* < 0.01, Table 4).

The miR-196a is an onco-miR reported to be frequently dysregulated in PDAC [27, 30]. Zhang et al. measured, when normalizing to U6, 0.35-1557-fold variations in tumor miR-196 expression [27]. In our tumors we determined a mean −2.2-fold decrease, when normalizing to U6 (*p* < 0.01, Table 7). Wang et al. demonstrated 16.05-fold increase in plasma samples with miR-16 as the endogenous control [30]. On the other hand, for miR-16, as well as for the alien spike or U91 we did not find significant differences in miR196a expression between cancer and normal tissues (*p* > 0.05, Table 4).

The miR-217 inhibits in vitro tumor cell growth and it functions as a potential tumor-suppressor by influencing the Akt/KRAS signaling pathway, therefore, miR-217 is frequently down-regulated in PDAC. MicroRNA miR-217 was down-regulated 10-fold in the study by Szafranszka et al., normalized relative to miR-24 [25]. However, Greither et al. determined only a mean −2-fold decrease with 18S as internal control [22]. Ma et al. demonstrated −3.91-fold decrease, using U6 for normalization [34]. On the other hand, Hong et al. found, that expression of miR-217 was down-regulated up to −62.5-fold in PDACs. They also used U6 like internal control [31]. In our samples, miR-217 expression was significantly down-regulated across all internal controls, with a maximum −24.39-fold decrease with U6 and a minimum −7.19-fold decrease with U6 + U91 combination (*p* < 0.01, Table 4).

Thus, for miRNAs with high positive or negative expression levels, such as miR-21, mir-155 or miR-217, fold change values may differ up to several times, depending on selected internal controls. Moreover, different internal controls can produce controversial results for miRNAs quantification, as it was demonstrated for miR-96, miR-148a or miR-196a.

### Comparing internal controls: U91 is a new endogenous control for microRNAs quantification in pancreatic cancer

RT-qPCR quantification of tumor miRNA expression may play an essential role in PDAC diagnostics, chemotherapy resistance and survival prediction. RT-qPCR-based experiments require endogenous controls for the result normalization, reliability and reproducibility. U6 small nuclear RNA [8–11, 14, 15, 27, 30, 37, 38], 18S [7] and 5S ribosomal RNAs [8, 15, 39, 40], small nucleolar RNAs RNU48, RNU43, RNU44 – commercial available Applied Biosystems assays [41], or miR-16 [30, 42, 43] were often used as the endogenous controls for miRNAs expression evaluation. However, there are data indicating, that expression levels of these reference genes may differ significantly in neoplastic and normal tissues [17–19]. This expression instability may introduce bias, when determining miRNA dysregulation in tumors. For example, U6 small nuclear RNA was the most common internal control [8–11, 14, 15, 27, 30, 38] for the quantification of miRNAs expression in PDAC. However, there are data, implying that U6 expression may be unstable in breast and cervical cancers [17, 19, 42]. Also, the amount of U6 may vary significantly in serum samples from patients with breast and colorectal cancers [18, 42]. According to our findings, U6 expression may show as high as an 8-fold difference in PDAC and normal pancreatic tissue (Table 8). On the other hand, U6 was determined as the second most stable gene by the NormFinder algorithm (Table 5). U6 also demonstrated greater expression stability in breast carcinoma tissue samples when compared with the snoRNAs RNU44, RNU48 and RNU43. Furthermore, changes in levels of these snoRNAs correlated with tumor morphology and patient prognosis [41]. However, U6 alongside 5S and miR-16 showed remarkable expression variability in tissue samples from patients with breast carcinoma [42].

The data about miR-16 expression in serum samples from the breast cancer patients are controversial. On the first look, this miRNA demonstrated significant expression variations [18, 42]. On the other hand, analysis with the geNorm algorithm has identified miR-16 as well as miR-425 as the most stable normalizers [43]. According to our measurements, expression of miR-16 varied significantly in pancreatic carcinomas (*p* < 0.01, Table 7). In addition, miR-16 was marked by the NormFinder algorithm as the least stable of the analyzed endogenous controls (Table 5).

Another possibility for RT-qPCR result normalization is the use of alien spike RNAs, such as miR-39 from C. elegans [18, 44], as internal controls. Also, these spike RNAs should be selected while taking into consideration that the same RNA sequences may already exist in the human genome. Surprisingly, according to the NormFinder analysis, the artificial spike was the least stable control (stability 0.085; Table 5). It must be taken into consideration, that the NormFinder algorithm can calculate variations between two groups including all normal and cancer samples, but it is unable to evaluate the differences between normal and cancer tissues from the individual patients. Accordingly, this may be the reason for the “instability” of the alien spike.

In this study, we compared the expression of 4 internal controls to determine the most stable of them. On the first look, the best internal control is the artificial spike, due to its amplification curves and threshold cycles, which have demonstrated to be very close for cancers and normal tissues (Table 7). On the other hand, according to the results, yielded by the NormFinder analysis, the best normalizer is the combination of U6 and U91. This combination has the best stability value, but as normalization results show, it differs significantly from the artificial spike (*p* < 0.01, Table 6). The most stable gene, determined by NormFinder, was U91 (Table 5). Each miRNA normalized relatively to the spike or U91, demonstrated similar expression values. Thus, statistically significant and insignificant differences between tumors and normal tissues for miRNAs were equal for the spike and the U91 (Table 4). Also, the differences between the spike and U91 were statistically insignificant for all of miRs except of miR-217 (Table 6). Among three endogenous controls, the U91 had the lowest average expression values and standard deviation in cancer tissues (Table 8).

Thus, we recommend U91 as a new normalizer of miRNA expression in pancreatic adenocarcinoma.

## Conclusions

We found expression of traditional endogenous controls, such as U6 and miR-16 can be unstable in pancreatic tumors and may vary up to several times. This instability may introduce bias to the miRNAs quantification. On the other hand, U91, the new stable internal control for miRNAs expression evaluation in pancreatic cancers was found.

## MIQE guidelines

This study was carried out in compliance to the Minimum Information for Publication of Quantitative Real–Time PCR Experiments (MIQE; [45]).

## Availability of data

Data files, including raw CT values or fold change tables are available on request, please, contact the correspondence author.

## Abbreviations

PDAC: Pancreatic ductal adenocarcinoma
RT-qPCR: Reverse transcription quantitative polymerase chain reaction
FFPE: Formalin-fixed, paraffin-embedded (tissues)
CT: Cycle threshold
